# Seventy years of publications

**DOI:** 10.1107/S205225251701168X

**Published:** 2017-09-01

**Authors:** S. Samar Hasnain

**Affiliations:** a Max Perutz Professor of Molecular Biophysics at the University of Liverpool and Editor-in-Chief of IUCr, Barkla X-ray Laboratory of Biophysics, Institute of Integrative Biology, Life Sciences Building, Liverpool, L69 7ZB, UK

**Keywords:** editorial, IUCr Journals

## Abstract

The IUCr, which was established 70 years ago, will be celebrating 70 years of peer-reviewed publications in 2018. Three of its journals, including *IUCrJ*, are ranked in the top 10% of all journals, with the remainder striving to be in the top quartile in the next five years.

One hundred years after the first Nobel prize to crystallography to Laue in 1914, the IUCr celebrated the impact and influence of crystallography in the International Year of Crystallography (IYCr2014) by launching a comprehensive, all-inclusive open-access journal *IUCrJ* with the aim of it becoming the ‘*PNAS* of the IUCr’. As we complete the first four years of publication and consider the papers that have appeared, the impact factors that have been achieved and the willingness of leaders of the wider community to submit their important papers to the journal, there are grounds to think that the ambition was not misplaced. Many of the papers that have been published have reported step changes in science with some of them reporting major advances in instrumentation and methods providing readers with an opportunity to see some excellent science in the chemical, materials and biological fields. The journal has become a natural home for the free-electron laser community, reporting many of the exciting developments and scientific breakthroughs in this area. We wish to encourage this further. Cryo-electron microscopy (cryoEM) is seeing a major transformation and has rightly been referred to as the ‘method of the decade’ in our columns. We will continue to work with the cryoEM community to make *IUCrJ* a natural choice for their important papers. The importance of cryoEM for structural science has been obvious to the IUCr for many years and was on display again at the 24th IUCr Congress held in August 2017 in Hyderabad.

*IUCrJ* aims to capture some of the important science and advances in methods and approaches presented at its Congresses. For the Hyderabad Congress, we are pleased to say that more than a dozen leaders have undertaken to contribute topical reviews, lead articles and research papers to the journal. Six of these have already appeared (Blundell, 2017 ▸; Braga *et al.*, 2017 ▸; Bryce, 2017 ▸; Hulliger *et al.*, 2017 ▸; Spence, 2017 ▸; Unwin, 2017 ▸). Several of the key papers from the Congress will be appearing in our specialist journals covering the whole range of topics from the foundations of crystallography to chemistry, materials and biology. Our aim is to work with the widest community as represented by the 21 commissions of the IUCr (http://www.iucr.org/iucr/commissions) and the broad beneficiaries of structural science as recognized by the award of 29 Nobel prizes (http://www.iucr.org/people/nobel-prize) in physics, chemistry, physiology and medicine. We also aim to ensure that *IUCrJ* and our other journals specializing in biology, chemistry, materials, and instrumentation and methods continue to provide fair and constructive peer review.

It is pleasing to see that in the latest impact-factor announcements three of our journals [*IUCrJ*, *Foundations and Advances* (*Acta Crystallographica A*) and *Structural Chemistry* (*Acta Crystallographica C*)] have been placed in the top 10% of over 12000 peer-reviewed journals, with *IUCrJ* and *Acta Crystallographica A* in the top 5%. The *Journal of Synchrotron Radiation*, established at the Beijing Congress in 1993, was ranked in the top 20%, with the *Journal of Applied Crystallography* at 26%. With increased engagement of the community and the hard work of our editors we aim to get most of our journals ranked in the top quartile. Please consider submitting some of your outstanding work to one of our nine journals. The science and technology they represent has been highlighted in recent IUCr flyers (see http://journals.iucr.org/services/promotions/promotions.html) entitled *IUCr Biology*, *IUCr Chemistry*, *IUCr Materials* and *IUCr Instrumentation and methods*. A dedicated *IUCrJ* flyer highlights 32 papers from 2016 and 2017 covering the whole range of sciences and technology. We encourage you to consider the journal alongside other notable journals such as *PNAS*, *JACS*, *Nature Communications* and *Nature Materials*. The scope and coverage of *IUCrJ* and our other journals will continue to evolve and expand in response to the changing landscape and the broader impact that our field and the Union is making. At the last Congress and General Assembly, several countries joined the IUCr and at the 24th Congress individuals have been offered the opportunity to become more closely involved with the Union through the Associates programme. We expect this, together with the science focus provided by our journals, will provide further cohesion as envisaged by the founders of the Union.

To mark the 70th anniversary of the Union and to attract scientific leaders from the wider structural science communities (both producers and users of structures) to join our journals, we issued two calls for editorial board members this spring. The first call resulted in the appointment of five Main Editors, namely Elena Boldyreva and Chiara Massera (*Crystallographic Communications*: *Acta Crystallographica E*), Ingolf Lindau (*Journal of Synchrotron Radiation*), Gary McIntyre (*Journal of Applied Crystallography*) and Mark van Raaij (*Structural Biology Communications*: *Acta Crystallographica F*). The second call, which primarily focused on Co-editorial positions, attracted highly qualified candidates and resulted in 19 new appointments with nine for our chemistry and materials journals, and nine for our structural biology journals, as well as one for *Foundations and Advances*: *Acta Crystallographica A*. This journal celebrates its 50th anniversary next year together with *Acta Crystallographica B*: *Structural Science, Crystal Engineering and Materials*. Next year also marks the 70th anniversary of *Acta Crystallographica*, the 25th anniversary of *Acta Crystallographica D*: *Structural Biology* and the 50th anniversary of the *Journal of Applied Crystallography*. It also marks the 25th anniversary of the formal approval by the Union’s General Assembly of the launch of *Journal of Synchrotron Radiation*; the approval of which came at a time when it was not obvious how major an impact synchrotron radiation would have on the crystallographic world. As we approach the 70th anniversary of peer-reviewed publications from the IUCr, we can take pride in our portfolio of publications and our aim of getting most of our journals into the top 20% of peer-reviewed publications with a number in the top 10%.

The quality of papers that we publish depends not only on the quality of submissions, but also on the fair and rigorous scientific peer review that our Editors, Co-editors and referees undertake. For the last two years papers in our journals have carried the name of the Co-editor responsible for handling the peer-review process for the paper. During 2018, we aim to give referees the choice of having their name published at the end of an article (or remaining anonymous), and hope that this will enable better recognition of the contribution that referees make to our journals.
